# The concept of disability and its causal mechanisms in older people over time from a theoretical perspective: a literature review

**DOI:** 10.1007/s10433-021-00668-w

**Published:** 2022-01-29

**Authors:** Ines Mouchaers, Hilde Verbeek, Gertrudis I. J. M. Kempen, Jolanda C. M. van Haastregt, Ellen Vlaeyen, Geert Goderis, Silke F. Metzelthin

**Affiliations:** 1 Department of Health Services Research, Faculty of Health Medicine and Life Sciences, CAPHRI Care and Public Health Research Institute, Maastricht University, P.O. Box 616, 6200 MD Maastricht, the Netherlands; 2 Living Lab of Ageing and Long Term Care, Maastricht, the Netherlands; 3 Department of Public Health and Primary Care, Academic Centre for Nursing and Midwifery, KU Leuven, Leuven, Belgium; 4 Department of Public Health and Primary Care, Academic Centre for General Practice, KU Leuven, Leuven, Belgium

**Keywords:** Disability, Ageing in place, Theory, Model, Person–environment fit, Older adults

## Abstract

Ageing with a disability increases the risk of hospitalization and nursing home admission. Ageing in place interventions aiming to reduce disability are often not sufficiently effective and inadequately theory-based. There are many models available on disability, but it is unclear how they define disability, what their differences are, and how they evolved throughout the years. This paper aims to provide an overview of the evolution of these models and to elaborate on the causal mechanisms of disability. A literature review was conducted as part of the TRANS-SENIOR international training and research network. PubMed and Google Scholar were searched, and snowball sampling was applied to eligible publications. Data were extracted from the included publications, and a thematic analysis was performed on the retrieved data. Overall, 29 publications were included in the final sample. All included models arose from three original models and could be divided into two types: linear models and models on the interaction between the person and the environment. Thematic analysis led to three distinct evolutionary trends: (1) from a unidirectional linear path to a multidirectional nonlinear path, (2) from the consequences of disease towards the consequences of person–environment interaction, and (3) from disability towards health and functioning. Our findings suggest that by optimizing the use of personal as well as environmental resources, and focusing on health and functioning, rather than disability, an older person’s independence and wellbeing can be improved, especially while performing meaningful daily activities in accordance with the person’s needs and preferences.

## Introduction

Increasing age is generally accompanied by an increased prevalence of disability. In the USA, for example, approximately 58.5% of the population aged 65 years and older suffer from disability, of whom 41.6% experience severe disability (U.S. Census Bureau 2018). In 2014, in the EU member states, 23.7% of the population aged 65 and over experienced a limitation in activities of daily living (ADL) (Eurostat 2020). Disability is defined as difficulty or dependency in carrying out ADL, mostly related to self-care and other activities that are essential to living independently (Fried et al. 2004; Lafortune and Balestat 2007). Disability is not simply a result of the person’s diminished abilities to perform ADL. A demanding social and physical environment can also stimulate or hinder participation in meaningful activities and the fulfilment of roles set by the person’s environment (de Vries et al. 2011). Ageing with a disability can lead to an accumulation of health risks, loss of independence, poor quality of life (QoL), and depression, which can in turn lead to an increased risk of hospitalization and (permanent) nursing home admission (Arnau et al. 2016; Tappenden et al. 2012). Consequently, this increased need for acute and long-term care is a challenge for society, which is already operating with financial and workforce constraints (Oliver et al. 2014), and makes the prevention of disability a key research topic (Cesari et al. 2015; Daniels et al. 2010).

Many older people also prefer to ‘age in place’ and thus remain in their own homes independently for as long as possible (Wiles et al. 2012). Over the last years, there has been a shift from residential to home-based care to meet the needs of the older population in a potentially more effective and financially sustainable way (Rostgaard et al. 2011). To support the ageing in place policy and avoid hospitalizations and (permanent) nursing home admission, various interventions have been developed aiming to promote daily functioning and reduce disability. However, current ageing in place interventions are not always sufficiently effective. One important reason for this seems to be that current interventions are inadequately theory-based, although theory has proven to be advantageous when developing effective interventions. Nonetheless, various studies on ageing in place interventions only refer loosely to theory rather than describing how theory helped inform the development process (Bartholomew and Mullen 2011; Michie and Prestwich 2010).

There are many models, theories and concepts (referred to as models from here on out) available on the onset and course of disability (Putnam 2003). It is, however, unclear how they define disability, what their differences are, and how these have evolved throughout the years. Therefore, there is a need for an overview of how theory explains the concept of disability, the onset of disability, and its causal mechanisms.

The aim of this paper is to (i) provide an overview of theoretical models explaining the concept of disability, (ii) gain insight in their development throughout the years, and (iii) elaborate on the causal mechanisms of disability in older people. This review could serve as a theoretical foundation for future interventions and policies aiming to reduce disability and its negative consequences in community-dwelling older adults, and ultimately promote ageing in place.

## Methods

For this paper, a literature review was conducted focusing on the analysis of theoretical models explaining the concept of disability throughout the years.

### Search procedure

This review combines two search techniques, electronic database search and snowball sampling, which were applied simultaneously. The search was performed between November 2019 and May 2020. Both scientific and grey literature were included.

#### Electronic database search

An electronic database search was performed to identify relevant literature using search terms such as ‘disability’, ‘disablement’, ‘person–environment fit’, combined with search terms such as ‘theory’, ‘theoretical framework’, ‘concept’, ‘conceptual framework’, ‘model’, and ‘conceptualization’. It was anticipated that the literature of interest would not only be found in regular databases; therefore, the search was conducted in PubMed (for scientific publications) and Google Scholar (for scientific and grey literature). There was no limitation regarding publication date because of the historical character of some key publications and the aim to map the evolution of the theoretical models included.

#### Snowball sampling

The snowball sampling technique was based on the guidelines for snowballing in systematic literature studies described by Wohlin (2014). Firstly, in order to identify the initial set of papers, we appealed to the expertise of the research team, who work in the field of social gerontology, public health and primary care, and long-term care. The papers they suggested in combination with the results of the electronic database search were used as a starting point for the review process. The next step was backward snowballing, where the reference lists were used to identify new papers to include. Next, to complete the results, PubMed and Google Scholar were used for forward snowballing, wherein new papers were identified based on papers citing the examined paper. This process was done until no new publications were identified.

### Selection procedure

#### Screening

An initial screening took place during which articles were screened for eligibility based on the title, and in cases where there was doubt, on the abstract as well. After this initial screening, eligible articles were again screened at title and abstract level to identify the publications that would potentially meet the inclusion and exclusion criteria as described below. If no abstract was available, for example in book chapters, the introduction or summary information provided by Google Scholar was used. Subsequently, the full-text of the selected publications was reviewed for eligibility. Screening was initially performed by one person (IM), after which the results of the screening process were discussed within the research team and adjusted based on consensus.

#### Inclusion and exclusion criteria

Publications were included when they: (a) developed and described a new model, theory, or concept (subsequently referred to as model) of disability, daily functioning (ADL and instrumental ADL), or person–environment fit, or analysed and refined an existing one; and/or (b) performed a literature review of one or more models in the domain of disability and contributed to the analysis of conceptual development of these models; and (c) were published in English. Publications were excluded when they did not contribute to the information retrieved from the original models and therefore did not add anything to the analysis of the theoretical models or their conceptual development over time (for example study protocols, or book chapters which merely provide a description of a theoretical model).

### Data extraction and analysis

After selection, data extraction was done using a data extraction spreadsheet in Microsoft Excel 2016 (Microsoft Corporation, Redmond, WA, USA). The following information was retrieved: (a) the goal of the publication, (b) the pathway or process described in the model, (c) the concepts defined and other elements included in this pathway, (d) strengths and shortcomings of the model identified during further development, and (e) underlying assumptions made on the causal mechanism of disability. Following data extraction, a thematic analysis was performed, during which results were grouped into themes. Eventually, possible trends in the evolution of these theoretical models were observed. This was an iterative process, during which results were reviewed, discussed and adjusted within the research team.

## Results

### Study characteristics

**Fig. 1:**
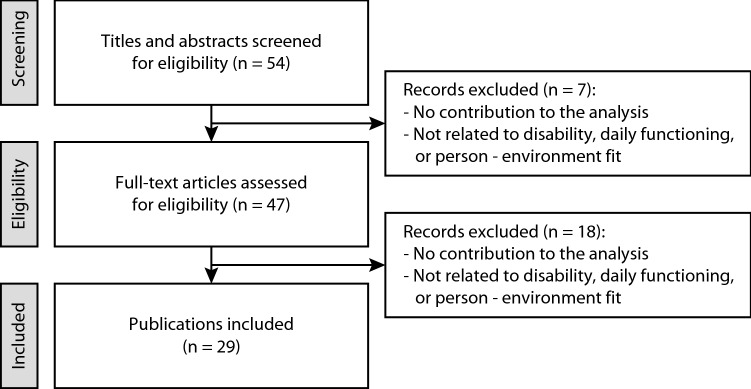
Flowchart of literature search process

After a prior screening of the literature, 54 titles and abstracts of core publications were screened. Of this final selection, 47 full-text articles were assessed for eligibility, resulting in 29 publications that met the inclusion criteria (Fig. 1). These 29 publications included 15 models and 14 literature reviews. Nine of the 29 publications were book chapters, two were government reports, one was a research report and the remaining 17 were scientific papers. The publications included were published between 1965 and 2018. The characteristics of all included publications are listed in Table 1.

**Table 1:** Characteristics and content of included publications

Theoretical models explaining the concept of disability	
Author (year)	Contribution
*Name (if applicable)*	
Nagi (1965) *Disablement Model*	• Describes a linear main pathway consisting of four distinct concepts: active pathology, impairment, functional limitation, and disability • Mainly focusing on the internal process of disability, without considering the role of the environment
Lawton and Nahemow (1973) *Competence–environmental press model*	• Presents the relationship between ageing individuals and their environment • The interaction between the individual’s competences and the pressure that is put upon the individual by the environment determines how the individual functions in that environment
World Health Organization (1980) (WHO) *ICIDH*	• Describes a linear main pathway consisting of four distinct concepts: disease, impairment, disability, and handicap • Mainly focusing on the internal process of disability, without considering the role of the environment
Kahana (1982) *Congruence model of person–environment interaction*	• Comment on Lawton and Nahemow’s model: a fit between the individual and their environment is based on both the environment’s characteristics and the individual’s preferences and needs, rather than their competences
Nagi (1991)	• Redefines the term disability as *‘an inability or limitation in performing socially defined roles and tasks expected of an individual within the sociocultural and physical environment’,* meaning it was not merely inherent in the individual • Lists several factors that could interfere with the links between different stages of the linear pathway (both individual characteristics, as well as the role of the individual’s social and physical environment and the individual’s reaction to this)
Pope and Tarlov (1991) (IOM)	• Adds risk factors to the ICIDH model, which could predispose the individual to disability. These factors could interfere with each stage of the main pathway • Adds QoL to the model as an integral part. QoL affects and is affected by the outcomes of each stage of the main pathway
National Center for Medical Rehabilitation Research (1993) (NCMRR)	• Extends the model presented by the IOM with societal limitations, defined as ‘*restrictions attributable to social policy or barriers which limit fulfilment of roles or deny access to services and opportunities associated with full participation in society’*
Verbrugge and Jette (1994) *The disablement process*	• Elaborates the linear pathway with Lawton’s environmental-press model. The main pathway is extended with personal and environmental factors that speed up or slow down disability by altering the demand of the environment or the capabilities of the individual
Brandt and Pope (1997) *Enabling – disabling process*	• Adds bidirectional arrows between the concepts of the main pathway described by Nagi, allowing the pathway to be reversed towards rehabilitation • Presents disability as an interaction of the individual with the environment and not solely an inherent part of the individual • Focusses on health and functioning and therefore, deletes the term ‘disability’ in the main pathway. The concept ‘no disabling condition’ is added at the beginning of the main pathway, indicating that there is also an ending to the pathway when no pathology, impairment, or functional limitation is present
Lawton (2000)	• Comments on the criticism of Kahana in 1982, and notes that the greater the competence of individuals, the more environmental resources are available to fulfil their needs and wishes
World Health Organization (2001) (WHO) *ICF*	• Provides a bidirectional and nonlinear representation instead of the linear main pathway. This allows for a more dynamic interaction between the individual’s functioning, and their health condition and environmental factors • Introduces different concepts: health condition, functions/structure, activity, and participation • Counters the view that people’s disability is a natural consequence of disease and presents a functional model instead of a medical model by including the positive aspects of functioning
Kahana et al. (2003)	• Extends the previous model from the institutional setting to the community setting
McDougall et al. (2010)	• Includes QoL in the graphical representation of the ICF as an outer subsystem around the original scheme, implying that it is incorporated in all aspects of functioning
Ravenek et al. (2013)	• Changes ‘health condition’ to ‘health’ in order to be all-inclusive • Presents the model as concentric circles, emphasizing the relationship between components and their potential interaction that takes place as part of human functioning • Presents human functioning as an interaction between body functions and structures, activities, and participation
Heerkens et al. (2018)	• Deletes the concept of ‘health’ and includes it in the component ‘personal factors’ as a (co)morbidity instead • Averts the emphasis from the biological components of the model by putting participation at the centre of the model

Literature reviews of one or more theoretical models	
Author (year)	Contribution
Kennedy and Minkler (1998)	• Highlights the lack of attention given to the role of the environment in both linear models presented by Nagi (1965) and the WHO (1980)
Jette and Badley (2000)	• Criticizes the linear models for failing to see disablement as a dynamic process that is not unidirectional or linear. The linear models view disabling conditions as a simple linear progression that is a response to diseases • Highlights the negative connotation of the ICIDH presented in 1980 by the WHO. The focus of this classification was on deficiencies resulting from health conditions
Nordenfelt (2003)	• Notes that the terms ‘disability’ and ‘handicap’ in the ICIDH are viewed as completely independent from the environment and the relationship between the person and his or her environment • Highlights two major changes made in the ICF: (1) includes positive aspects of functioning, and (2) grants a crucial role to the environment in this classification
Ustün et al. (2003)	• Criticized the ICIDH for being too focused on the disabilities, rather than being a neutral classification of human functioning • Notes the lack of personal and environmental factors in the progression towards handicap throughout the ICIDH • States that the ICF combines the medical model of disability with the social model, meaning that disability is a combination of something inherent in the individual as well as a socially created problem due to an unaccommodating social environment
Schneidert et al. (2003)	• Criticizes the ICIDH for its limited role of the environment and its focus on the individual in the path of disability
Scheidt and Norris-Baker (2004)	• Elaborates on the development of Lawton and Nahemow’s competence–environmental press model after criticism received by Kahana (1982) on the lack of inclusion of needs and preferences of the individual
Heikkinen (2006)	• Notes the linear and unidirectional character of Nagi’s Disablement Model where the main pathway is assumed a sequence of events leading towards disability
Whiteneck (2006)	• Criticizes the ICIDH for not incorporating the role of environmental factors into the classification • States that the ICF improved by including environmental factors into their classification • Criticizes the ICF for not distinguishing ‘activities’ and ‘participation’ enough from each other. ‘Activities’ needs to be defined as something on the individual’s level, whereas ‘participation’ needs to be defined as something on a societal level
Nordenfelt (2006)	• States that the focus of the ICIDH was mainly on the tasks individuals are unable to do because of diseases or injuries
Jette (2006)	• Criticizes the linear models for failing to see disablement as a dynamic process that is not unidirectional or linear. The linear models view disabling conditions as a simple linear progression that is a response to diseases
Masala and Petretto (2008)	• Emphasizes the lack of acknowledgement of the role played by the environment in the linear models of Nagi and the WHO • Highlights the improvements Nagi made to his Disablement Model in 1991, namely the recognition of the role of the characteristics of the individual and the environment in the disablement process • Notes that the role of the environment described in Nagi (1991) only refers to the demand it puts on an individual and therefore, disability is still viewed as part of the individual • Criticizes the model presented by the IOM in 1991 for its linearity without the possibility of reversing in the pathway • Criticizes the model presented by the IOM in 1991 for the limited role played by the environment, especially the social environment. The environment is only included as a risk factor instead of progression in the pathway
Iecovich (2014)	• Criticizes the competence–environmental press model for not taking into account the individual’s attributes. The model does not acknowledge that the individual can manipulate the environment to reduce the press, or that they can use the environment as a resource to fulfil their needs and wishes • Highlights that the competence–environmental press model does not provide strategies to measure person–environment linkages
Petretto et al. (2017)	• Highlights the importance of the ICF in terms of shifting the focus away from disability being a static event, towards a dynamic process that may vary over a life course
Vaz et al. (2017)	• Criticizes Nagi’s Disablement Model and the ICIDH for their linear and unidirectional character, implying that a pathology triggers the disabling process consisting of stepwise negative consequences. This also highlights that the focus is on the disease and the negative consequences instead of health and functioning • Criticizes both linear models for being too organism-limited, meaning that they fail to identify the role played by the environment during this process • Highlights the major improvements made when developing the ICF: • The interactive and bidirectional character of the ICF emphasizes that the nature of disability and functioning lies within the interaction between health conditions and contextual factors; • The ICF counters the medical point of view, which states that disability is a natural consequence of diseases; • The ICF considers different influences on health and functioning (biological, individual, and social factors)

*ICIDH* = International Classification of Impairments, Disabilities, and Handicap; *ICF* = International Classification of Functioning, Disability, and Health; *IOM* = Institute of Medicine; *NCMRR* = National Center for Medical Rehabilitation Research; *QoL* = Quality of Life; *WHO* = World Health Organization

### Three original models

**Fig. 2:**
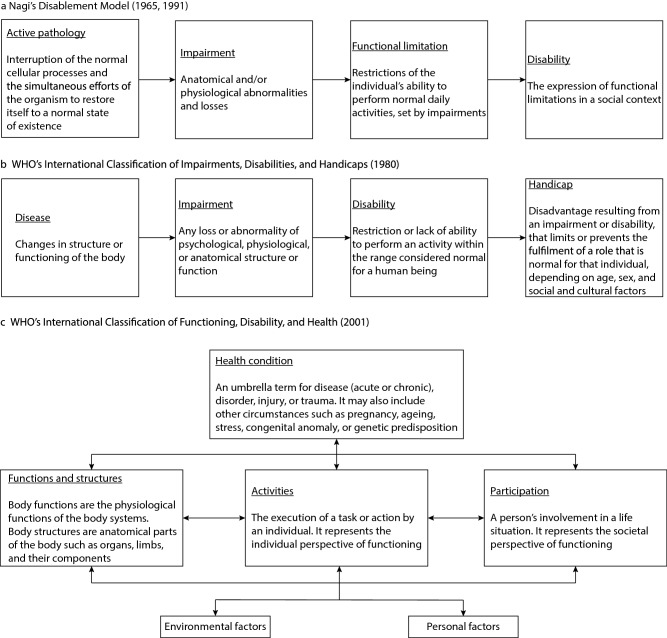
(**a**) Graphical representation of Nagi’s Disablement Model (Nagi 1965, 1991), (**b**) the WHO’s ICIDH, and (**c**) the WHO’s ICF. Figure 2b and 2c are adapted with permission from the World Health Organization (1980, 2001)

Three original models could be identified throughout the 29 included publications; 1) Nagi’s Disablement Model, 2) the WHO’s International Classification of Impairments, Disabilities, and Handicaps, and 3) Lawton’s competence–environmental press model. These original models are described below. First, in the 1960s, sociologist Saad Nagi described the process of disablement as the Disablement Model. To provide clarity on the terms and concepts surrounding disability, Nagi described the process of disablement as a linear main pathway consisting of four distinct stages: active pathology, impairment, functional limitation, and disability (Fig. 2a) (Nagi 1965). Second, in 1980, the World Health Organization (WHO) published the International Classification of Impairments, Disabilities, and Handicaps (ICIDH). Similar to Nagi’s Disablement Model, this conceptual framework follows a linear main pathway, consisting of four concepts that classify the consequences of disease and their implications for the lives of individuals (Fig. 2b) (World Health Organization 1980). In 2001, the WHO revised its classification and developed the International Classification of Functioning, Disability and Health (ICF) (World Health Organization 2001). This revision is considered a major turning point in the evolution of disability models as it is the first model to attempt to address all of the ICIDH’s major shortcomings. These shortcomings and how the ICF addressed them are described in the section later on. The new classification also introduced the terms ‘health condition’, ‘functions/structure’, ‘activity’, and ‘participation’ in their presentation next to the environmental and personal factors (Fig. 2c) (World Health Organization 2001). Moreover, the ICF presents the individual’s functioning as a dynamic, bidirectional, and nonlinear interaction between an individual’s health condition and environmental factors (Vaz et al. 2017; World Health Organization 2001). It is therefore the most widely accepted model of functioning and disability (Vaz et al. 2017). The third original model is Lawton’s competence–environmental press model, described in the 1970s, which presents the relationship between ageing individuals and their environment. This model conceptualizes the individual as having a set of competences and the environment putting pressure upon that individual. Both the individual competences and environmental pressures may fluctuate over time, resulting in positive or negative effects (Lawton and Nahemow 1973). The interaction between both individual competences and environmental pressure determines the extent to which individuals are able to function optimally in that environment. To achieve a good fit between the personal competences and the environmental pressure, it is possible to interfere with both factors (Iecovich 2014; Lawton and Nahemow 1973).

Based on these three models, two types of models can be distinguished. First, Nagi’s Disablement Model and the WHO’s ICIDH present a clear linear pathway build of distinct concepts. Both models generally describe the same process but use different terms to do so; for example, Nagi uses functional limitations to describe a restriction of the individual’s abilities to perform activities of daily living, whereas the WHO uses the term ‘disability’ to define these restrictions. Additionally, both models mainly focus on the internal process of disability related to the person themselves and do not consider the role of the outer physical and social environment (Vaz et al. 2017). Secondly, there is Lawton’s competence–environmental press model presenting the relationship between the individual and the environment, and thus, focusing on more than solely the internal process of disability.

### Disability over time

**Fig. 3:**
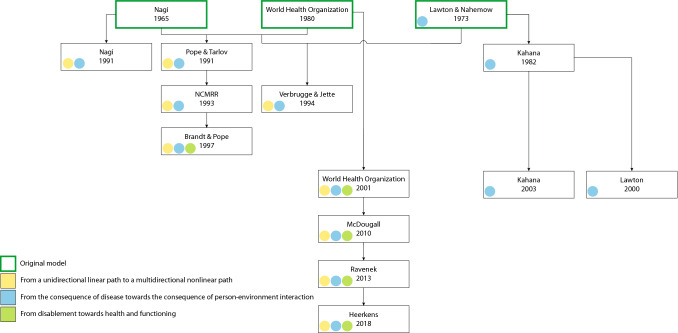
Flowchart presenting the development of theoretical models explaining the concept of disability throughout the years. The figure also presents the results from the thematic analysis; three main trends throughout the evolution: (1) from a unidirectional linear path to a multidirectional nonlinear path (yellow), (2) from the consequence of disease towards the consequence of person–environment interaction (blue), and (3) from disablement towards health and functioning (green). The colour code indicates which evolutionary trend is represented in this model, either by a visual representation or in the description. The three models at the top present the original models (green frame). NCMRR stands for National Center for Medical Rehabilitation Research

Over the years, these three models underwent further development following progressive insights into disability and its underlying causal mechanisms. To elaborate on these developments and new insights, a thematic analysis was performed, identifying three trends: (1) from a unidirectional linear path to a multidirectional nonlinear path, (2) from the consequence of disease towards the consequence of person–environment interaction, and (3) from disablement towards health and functioning. Figure 3 presents the evolution of the models throughout the year.

#### 1) From a unidirectional linear path to a multidirectional nonlinear path

The static, linear, and unidirectional presentation of both Nagi’s Disablement Model and the ICIDH was considered overly simplistic (Heikkinen 2006; Vaz et al. 2017; World Health Organization 1980). This unidirectional, linear presentation claims that the presence of a pathology or disease initiates the disablement process with its stepwise negative consequences as an inevitable and irreversible result (Heikkinen 2006; Jette 2006; Jette and Badley 2000; Vaz et al. 2017). These insights led to several revisions of the unidirectional, linear pathway (Fig. 3, yellow dots). At first, authors mentioned the issue of the unidirectional process and that there were possibilities to return in the pathway, but this was never addressed in the graphical presentation (Nagi 1991; National Center for Medical Rehabilitation Research 1993; Pope and Tarlov 1991; Verbrugge and Jette 1994). The unidirectional and linear presentation was only addressed in later revisions by Brandt and Pope (1997) and the World Health Organization (2001).

By adding bidirectional arrows between the concepts of the linear pathway, Brandt and Pope (1997) allow the pathway to be reversed towards rehabilitation. This is important to acknowledge since it clarifies that disability is not necessarily an end stage. With the correct treatment or interventions, this pathway can be halted or even reversed. The WHO not only addressed the unidirectional but also the linear character of the models when developing the ICF (Fig. 2c) (World Health Organization 2001). The ICF does not present the ‘process’ of functioning and disability, but rather presents the individuals’ functioning as a dynamic, multidirectional and nonlinear interaction between an individual’s health condition and environmental factors (for example, the attitudes of the society, architectural characteristics, the legal system) (Petretto et al. 2017; Vaz et al. 2017; World Health Organization 2001).

#### 2) From the consequence of disease towards the consequence of person–environment interaction

Early models described disability mainly as a consequence of diseases (Nagi 1965; World Health Organization 1980). Models adhering to this principle described the disablement process as something that is unleashed from pathology or disease and runs through different stages until it results in disability (Vaz et al. 2017). However, when looking into the causal mechanism of disability, it was noted that disability is not inherent in the individual. Therefore, it was concluded that the early linear models lacked a construct to identify the role played by the environment in this process (Kennedy and Minkler 1998; Masala and Petretto 2008; Nordenfelt 2003; Schneidert et al. 2003; Ustün et al. 2003; Vaz et al. 2017; Whiteneck 2006). This led to several revisions and further development of both linear models, being Nagi’s Disablement Model and the WHO’s ICIDH (Fig. 3, blue dots).

In a first attempt to acknowledge the role played by the environment on the process of disability, risk factors were added that could predispose the individual to disability (Nagi 1991; Pope and Tarlov 1991; Verbrugge and Jette 1994). However, the contribution of the environment was still considered limited in the influence it had on the possible progression in the pathway instead of interacting with the individual (Petretto et al. 2017). Two models attempted to make the disablement process more of a social construct, meaning it was not solely related to the physiological and physical state of the individual, but how these restrictions present themselves in society (Nagi 1991; National Center for Medical Rehabilitation Research 1993).

It was not until later that disability and functioning were seen as an interaction between the individual and the environment they reside in (Brandt and Pope 1997; World Health Organization 2001). This means that the environment will play a critical role in the outcome of the person’s health condition and degree of functioning since an individual will experience greater disability in a less supportive environment than they would in a more supportive environment or context (Brandt and Pope 1997). The support can come either from the physical environment or from the social environment, making disability and functioning a social construct that is holistic and not only inherent in the individual (Brandt and Pope 1997; World Health Organization 2001).

In contrast to linear models, the model of Lawton and Nahemow (1973) already addressed the environmental influence early on. This model was also revised several times. In 1994, Verbrugge and Jette integrated the competence–environmental press model to elaborate the linear models to a full sociomedical concept (Verbrugge and Jette 1994). Both Lawton and Nahemow (1973) and Verbrugge and Jette (1994) indicate that the process of disability can be altered on both the individual level as well as the environmental level by changing the demand of the environment or the capabilities of the individual. This is highly important when designing interventions aiming to reduce or overcome disability. These interventions should aim for a perfect fit between the individual and the environment where they reside (Iecovich 2014). Later, it was emphasized that a fit between individuals and their environment is based on the environment’s characteristics and the individual’s preferences and needs, rather than their competences and that the environment provides resources and opportunities for the individual instead of only demanding something from the individual (Iecovich 2014; Kahana 1982; Kahana et al. 2003; Lawton 2000; Scheidt and Norris-Baker 2004).

#### 3) From disablement towards health and functioning

During the evolution of the linear models, it was considered important to divert the focus away from the person’s disability and what they were no longer able to do, and instead to focus on health and functioning, as well as participation in a community and a person’s wellbeing (Jette and Badley 2000; Nordenfelt 2006; Ustün et al. 2003; Vaz et al. 2017). As shown in Fig. 3 (green dots), this focus only changed after several revisions. Brandt and Pope (1997) attempted to tailor the model towards rehabilitation by creating the possibility to return to a state of ‘no disabling condition’. By introducing new terms into their dynamic model, the WHO included the positive aspects of functioning, which was considered being a major improvement from the ICIDH (Nordenfelt 2003; World Health Organization 2001). A major step forward in averting the focus towards health and functioning was made in the ICF. In this classification, they introduced the terms ‘health condition’, ‘functions/structure’, ‘activity’, and ‘participation’ in their presentation next to the environmental and personal factors described earlier (Fig. 2c) (World Health Organization 2001). By introducing these terms, they included the positive aspects of functioning, and focused on the role someone plays or wishes to play in the community.

Several authors tried to build on the graphical representation of the ICF after some minor shortcomings were defined (Heerkens et al. 2018; McDougall et al. 2010; Ravenek et al. 2013), their contributions are listed in Table 1. For example, the model does not include the subjective experience of health or quality of life. It is, however, important to consider the person’s wellbeing when assessing health and functioning (McDougall et al. 2010; Ravenek et al. 2013; Whiteneck 2006). Additionally, the term ‘health condition’ might be confusing as it may imply that there is a condition to consider, therefore making the scheme not universally applicable since there are also people without any conditions (Heerkens et al. 2018; Ravenek et al. 2013). Lastly, the graphical representation emphasizes ‘health conditions’ because it is put at the top of the scheme, giving priority to the biological components of the model (Heerkens et al. 2018; Ravenek et al. 2013). Despite these shortcomings and the attempts to overcome these, the ICF remains the most widely accepted model in terms of disability, health, and functioning (Vaz et al. 2017).

## Discussion

This literature review provided an overview of theoretical models explaining the concept of disability, provided insight into their development throughout the years and elaborated on the causal mechanisms of disability in older people. The results showed that all models originate from three models: Nagi’s Disablement Model, the WHO’s ICIDH, and Lawton and Nahemow’s competence–environmental press model. These early models can be classified into two types, the linear models on the one hand (Nagi 1965; World Health Organization 1980) and a model focusing on the interaction between the individual and their environment on the other hand (Lawton and Nahemow 1973). All three of these models were further developed throughout the years, resulting in an evolution from a medical to a biopsychosocial concept. Throughout this evolution, there were three clear trends visible: (1) from a unidirectional linear path to a multidirectional nonlinear path, (2) from the consequence of disease to the consequence of person–environment interaction, and (3) from disablement towards health and functioning. Analysing these themes led to a better insight into the causal mechanisms of disability in older people, and how these should be considered and applied in current practices. These insights are especially important when aiming to promote ageing in place and avoid unnecessary care transitions.

The results of this review indicate that disability is not a static concept, but a dynamic and interactive process that may fluctuate over a life course. Moving away from the linear and unidirectional graphical presentation of disability and functioning has several advantages. Firstly, it reflects the fact that disability is not automatically an end stage but can be reversible. Secondly, the more dynamic and nonlinear concept of disability reflects the opportunities of individuals to move away from a disabled state supported by interventions or other influences. This is supported by the results of Whitehead et al. (2015) and Resnick et al. (2013) both performed a literature review of interventions aiming to maintain or optimize functional abilities and reduce dependency in ADL. Both concluded that these interventions were effective in improving functional abilities (Resnick et al. 2013; Whitehead et al. 2015).

Additionally, changing the medical perspective of disability to a more social and integrated perspective adequately reflects the individual as part of an environmental context or a community, wherein they function. Obtaining an optimal fit between the individual and their environment is key for this individual to function adequately in this environment and can contribute to the individual’s wellbeing. Since adequate functioning and wellbeing are determined by the interaction between the individual and the environment, both can be targeted when designing interventions aiming to reduce disability and promote ageing in place. It is important to note that the environment can be seen as both a resource to support the individual in daily life and a burden to the individual’s functional state. For example, an older adult living in a deprived neighbourhood with few services and a poorly accessible built environment will face barriers to his or her mobility and social participation on a daily basis. However, when this person would live in a neighbourhood with more services close by, for example a community centre, the person would have access to a broader social network whose support might compensate the burden of the poorly accessible built environment. Moreover, going to the community centre could encourage the person to push the boundaries of their functional state. The concept of person–environment fit is also reflected in the WHO’s Healthy Ageing framework. Healthy Ageing is described as ‘*the process of developing and maintaining the functional ability that enables well-being in older age*’ (World Health Organization 2015)’. Functional ability is the interaction between intrinsic capacity, defined by genetic, personal and health characteristics, and the environment. Healthy ageing is something that can be achieved for everyone, where ‘healthy ageing’ is not viewed as the absence of a disease, but is seen as fostering an individual’s functional ability to be and do what they value (World Health Organization 2020). When aiming for ageing in place, an environment should be created in which the individual can age safely and independently, in line with their intrinsic capacity. This principle was described by the WHO as ‘age-friendly environments’ which aim to encourage active and healthy ageing by optimizing health, stimulating inclusion and enabling wellbeing in older age by adapting physical and social environments and municipal services to the needs and wishes of older people with varying capacities (World Health Organization 2017). The Homestead Care Model described by de Boer et al. (2021) is an illustrative example of how care facilities can develop age-friendly environments. It aims to provide opportunities for senior citizens for an active, meaningful daily life. It enables senior citizens to be part of society by ensuring a congruent physical, social, and organizational environment (de Boer et al. 2021). It should be noted that ageing in place may be experienced differently in different countries and cultural environments due to differences in health care systems, health policies, and access. This indicates that it requires a non-uniform approach tailored to the specific circumstances in different countries or regions (Aspinal et al. 2016). Other personal factors can also influence the individual’s capacity to age in place, for example the social network or socio-economic status (Bosch-Farré et al. 2020; Pani-Harreman et al. 2021). Furthermore, it is considered important to acknowledge the individual’s needs and wishes alongside their competences. This is related to the construct of agency, which is an individual’s capacity to make their own decisions related to future plans (Romaioli and Contarello 2019).

Lastly, changing the perspective towards health and functioning, rather than disability, reflects the current focus on wellbeing and positive aspects of individuals’ health. Not only does this emphasize what a person is able to do, it also reflects the opportunity to focus on what they would like to do in their own social and physical environment. When aiming to support the person to age in place, it is necessary to consider their capabilities and emphasize these, rather than only focusing on their limitations in daily life. This will ultimately contribute to a person’s wellbeing and quality of life. This is supported by Huber et al. (2011), who proposed a new concept of social health, which is characterized by having the capacity to fulfil one’s potential and obligations; the ability to manage life with some degree of independence, despite a medical condition; and participation in social activities. Based on this concept of social health, Dröes et al. (2017) proposed the operationalization of this concept in people living with dementia, together with an overview of factors and interventions that could influence or improve social health in this population.

The ICF is, despite its shortcomings, still the most widely accepted model regarding disability, health, and functioning, meaning that the overall perspective of disability being a dynamic interaction between an individual’s health and the environment where they reside in has not changed for over a decade (Vaz et al. 2017; World Health Organization 2001). This has had several consequences for policy and treatment. For example, nowadays many countries stimulate an ageing in place policy to enable older individuals to remain at home independently for as long as possible (Beard et al. 2016; Rostgaard et al. 2011). Ultimately, this translates to several interventions aiming to encourage independent living at home. However, many interventions only focus on either the individual or the environment and not the ageing individual as part of their physical and social environment. An intervention that aims to incorporate both is the CAPABLE intervention, which has proven its effectiveness with regard to (I)ADL disability scores (Szanton et al. 2019). The CAPABLE intervention targets both modifiable intrinsic (person-based) as well as extrinsic (environmental-based) factors that contribute to disability to achieve clients’ individual goals (Szanton et al. 2019). Even the CAPABLE intervention, despite the wide implementation, only focusses on the person’s individual capabilities and the optimization of the home environment without inclusion of the community or social environment (Szanton et al. 2021). Providing interventions that address both the individual and the environment requires new roles and tasks of professionals, strong collaboration among all partners involved in the care process and funding models that facilitate the new way of working. Consequently, despite attempts to shift the focus towards this new perspective, it remains a challenge to change existing organizational structures and achieve successful behavioural change of health care professionals (Ajani and Moez 2011; Reay et al. 2021). Ultimately, this also explains why currently there is still a big gap between theory and practice. Translating theory in a practical real-world setting is often challenging (Ajani and Moez 2011) and requires time and effort of many stakeholders in healthcare, which possibly explains the lack of theoretical foundation when developing interventions aiming to reduce disability and promote ageing in place.

### Limitations and strengths

One of the limitations of this literature review is that only two databases were used to complete the literature search. Therefore, it is possible that some key publications were missed. However, this risk was limited by consulting the research team during the search process and the use of snowballing. An additional limitation is that the scientific substantiation of the included publications was not systematically analysed, and no quality assessment was performed. However, nearly all included theoretical models are widely acknowledged by the research team, who are considered experts in the field.

Notwithstanding these limitations, this literature review contributes to the current literature by providing an all-inclusive overview that also presents the development of these models throughout the years, which to our knowledge has never been done before. Additionally, the literature review provides insights into the causal mechanisms of disability and translate this into practical implications for future research and practices for supporting the upcoming ageing in place policy. This literature review may serve as a theoretical foundation and rationale for multiple future initiatives.

### Implications

Despite the ICF being the most widely accepted classification of functioning, disability and health until today, it dates from 2001 and literature has proven that it has some shortcomings. To our knowledge, no attempt to improve and further develop this classification has been picked up in international literature to become the newest, most accepted theoretical model regarding functioning, disability, and health. Therefore, there is a possibility for future research to address these shortcomings when developing new theoretical models. For instance, the ICF in its current form is not universally applicable because the term ‘health condition’ implies that people without any conditions are not considered in this classification. Additionally, the classification does not include a subjective perception of health and functioning, such as wellbeing and quality of life. These shortcomings emphasize that the ICF is still a classification, which is mainly focused on functioning and more specifically on the restrictions and limitations individuals experience when performing an activity or during participation in a social role.

Future revisions of the ICF model should attribute sufficient attention to the abilities and strengths of the individual, how these can be enhanced or supported, and how they contribute to the person’s wellbeing and quality of life. Furthermore, it should become a universally applicable model that does not exclude people without any health conditions. When considering how these abilities and strengths can be promoted, both individual factors (such as age, gender, social status, etc.) and environmental factors, which is not only the built and physical environment, but also the social environment and support network of the individual, should be considered. In fact, the social environment plays an important role in the perception and acceptance of having a disability and how one deals with such disability. The theoretical model should provide a base for the development of future interventions or programs to support older people in their daily life. However, the assumptions made by the theoretical model regarding these strategies and implications need to be sufficiently supported by scientific evidence.

The current research also provides several important implications for future initiatives that aim to support the ageing in place policy and avoid hospitalizations and (permanent) nursing home admission. Firstly, it is essential to not only focus on treating disease, but also on the person’s capabilities. Treating the diseases alone is not the correct strategy to support a person to remain living at home independently; there also needs to be more attention to what the person is still able to do and how to highlight this during meaningful daily activities. However, treating underlying diseases remains important. If these diseases are left untreated, strengthening the person’s capabilities will become more difficult and progression can be undone quickly.

Secondly, when emphasizing the person’s capabilities during meaningful daily activities, both the person and their environment should be considered as resources to do this. This can be done in several ways. Firstly, one can use the current environment to support the person during their daily activities, for example by learning to use public transportation so that the person can travel longer distances independently, which enhances the participation of the person in the community. Secondly, one can alter the environment in such a way that it becomes more supportive for the person, for example installing grab bars in the bathroom.

Thirdly, individual support or treatment plans should be tailored to the person’s preferences, needs, and wishes, rather than his or her health condition. Treating underlying diseases, enhancing physical functioning, and promoting independence during meaningful daily activities alone is not sufficient for a person to remain at home independently. It is highly important to also focus on the wellbeing and quality of life of the individual and to do this it is necessary to adhere to what the person really wants in life and to what they draw energy from.

Lastly, following the implications mentioned above, it is important to consider the right outcome measures when evaluating an intervention or programme that supports the ageing in place policy and possibly avoid hospitalizations and (permanent) nursing home admission. As indicated previously, functional outcome measures such as ADL or physical performance are useful to consider and are currently the most used evaluation measures. However, quality of life and participation measures should also have an important role in the evaluation of such programmes. Ultimately, the goal is not only to remain living at home independently for as long as possible, but also to experience a good quality of life. This goes beyond physical performance and independent functioning.

## References

[CR1] Ajani K, Moez S (2011). Gap between knowledge and practice in nursing. Procedia Soc Behav Sci.

[CR2] Arnau A, Espaulella J, Serrarols M, Canudas J, Formiga F, Ferrer M (2016). Risk factors for functional decline in a population aged 75 years and older without total dependence: a one-year follow-up. Arch Gerontol Geriatr.

[CR3] Aspinal F, Glasby J, Rostgaard T, Tuntland H, Westendorp RG (2016). New horizons: Reablement – supporting older people towards independence. Age Ageing.

[CR4] Bartholomew LK, Mullen PD (2011). Five roles for using theory and evidence in the design and testing of behavior change interventions. J Public Health Dent.

[CR5] Beard JR, Officer A, de Carvalho IA, Sadana R, Pot AM, Michel J-P, Chatterji S (2016). The World report on ageing and health: a policy framework for healthy ageing. The Lancet.

[CR6] Bosch-Farré C, Malagón-Aguilera MC, Ballester-Ferrando D, Bertran-Noguer C, Bonmatí-Tomàs A, Gelabert-Vilella S, Juvinyà-Canal D (2020) Healthy ageing in place: enablers and barriers from the perspective of the elderly. A qualitative Study. Int J Environ Res Public Health, 17(18). 10.3390/ijerph17186451 10.3390/ijerph17186451 PMC7559318 32899744

[CR7] Brandt EN, Pope AM, Brandt EN, Pope AM (1997). Models of disability and rehabilitation. Enabling America: assessing the role of rehabilitation science and engineering.

[CR8] Carnemolla P, Bridge C (2020). A scoping review of home modification interventions – Mapping the evidence base. Indoor Built Environ.

[CR9] U.S. Census Bureau. (2018). Americans With Disabilities: 2014. Retrieved from https://www.census.gov/content/dam/Census/library/publications/2018/demo/p70-152.pdf

[CR10] Cesari M, Vellas B, Hsu, F-C, Newman AB, Doss H, King AC, Group LS (2015). A physical activity intervention to treat the frailty syndrome in older persons-results from the LIFE-P study. J Gerontol A Biol Sci Med Sci.

[CR11] Chase CA, Mann K, Wasek S, Arbesman M (2012). Systematic review of the effect of home modification and fall prevention programs on falls and the performance of community-dwelling older adults. Am J Occup Ther.

[CR12] Daniels R, Metzelthin S, van Rossum E, de Witte L, van den Heuvel W (2010). Interventions to prevent disability in frail community-dwelling older persons: an overview. Eur J Ageing.

[CR13] de Boer B, Bozdemir B, Jansen J, Hermans M, Hamers JPH, Verbeek H (2021) The homestead: developing a conceptual framework through co-creation for innovating long-term dementia care environments. Int J Environ Res Public Health 18(1):57 10.3390/ijerph18010057 PMC7795205 33374761

[CR14] de Vries NM, Staal JB, van Ravensberg CD, Hobbelen JSM, Olde Rikkert MGM, Nijhuis-van der Sanden MWG (2011). Outcome instruments to measure frailty: a systematic review. Ageing Res Rev.

[CR15] Dröes RM, Chattat R, Diaz A, Gove D, Graff M, Murphy K, Charras K (2017). Social health and dementia: a European consensus on the operationalization of the concept and directions for research and practice. Aging Ment Health.

[CR16] Eurostat (2020) Disability statistics - Elderly needs for help or assistance. Retrieved from https://ec.europa.eu/eurostat/statistics-explained/index.php?title=Disability_statistics_-_elderly_needs_for_help_or_assistance

[CR17] Fried LP, Ferrucci L, Darer J, Williamson JD, Anderson G (2004). Untangling the concepts of disability, frailty, and comorbidity: implications for improved targeting and care. J Gerontol A Biol Sci Med Sci.

[CR18] Heerkens YF, de Weerd M, Huber M, de Brouwer CPM, van der Veen S, Perenboom RJM, van Meeteren NLU (2018). Reconsideration of the scheme of the international classification of functioning, disability and health: incentives from the Netherlands for a global debate. Disabil Rehabil.

[CR19] Heikkinen E (2006). Disability and physical activity in late life - Rresearch models and approaches. Eur Rev Aging Phys Act.

[CR20] Huber M, Knottnerus JA, Green L, Horst H, Jadad AR, Kromhout D, Smid H (2011). How should we define health?. BMJ.

[CR21] Iecovich E (2014). Aging in place: from theory to practice. Anthropological Notebooks.

[CR22] Jette AM (2006). Toward a common language for function, disability, and health. Phys Ther.

[CR23] Jette AM, Badley E (2000) Conceptual issues in the measurement of work disability. In: N. Mathiowetz & GS Wunderlich (Series Eds.), Survey measurement of work disability: Summary of a workshop (pp. 4–27). doi: 10.17226/9787 25077260

[CR24] Kahana E, Lawton MP, Windley PG, Byerts TO (1982). A congruence model of person-environment interaction. Aging and the environment: theoretical approaches.

[CR25] Kahana E, Lovegreen L, Kahana B, Kahana M (2003). Person, environment, and person-environment fit as influences on residential satisfaction of elders. Environ Behav.

[CR26] Kennedy J, Minkler M (1998). Disability theory and public policy: implications for critical gerontology. Int J Health Serv.

[CR27] Lafortune G, Balestat G (2007) Trends in severe disability among elderly people: assessing the evidence in 12 OECD countries and the future implications. OECD Health Working Papers (26). 10.1787/217072070078

[CR28] Lawton MP, Birren JE, Schroots JJF (2000). Chance and choice make a good life. A history of geropsychology in autobiography.

[CR29] Lawton MP, Nahemow L (1973). Ecology and the aging process. The psychology of adult development and aging.

[CR30] Lette M, Ambugo EA, Hagen TP, Nijpels G, Baan CA, de Bruin SR (2020). Addressing safety risks in integrated care programs for older people living at home: a scoping review. BMC Geriatr.

[CR31] Masala C, Petretto DR (2008). From disablement to enablement: conceptual models of disability in the 20th century. Disabil Rehabil.

[CR32] McDougall J, Wright V, Rosenbaum P (2010). The ICF model of functioning and disability: incorporating quality of life and human development. Dev Neurorehabil.

[CR33] Michie S, Prestwich A (2010). Are interventions theory-based? development of a theory coding scheme. Health Psychol.

[CR34] Nagi SZ, Sussman MB (1965). Some conceptual issues in disability and rehabilitation. Sociology and Rehabilitation.

[CR35] Nagi SZ (1991). Appendix A: Disability concepts revisited: Implications for prevention. Disability in america: Toward a national agenda for prevention.

[CR36] National Center for Medical Rehabilitation Research (1993). Research plan for the National Center for Medical Rehabilitation Research (NCMRR).

[CR37] Nordenfelt L (2003). Action theory, disability and ICF. Disabil Rehabil.

[CR38] Nordenfelt L (2006). On health, ability and activity: Comments on some basic notions in the ICF. Disabil Rehabil.

[CR39] Oliver D, Foot C, Humphries R (2014) Making our health and care systems fit for an ageing population Retrieved from https://www.kingsfund.org.uk/publications/making-our-health-and-care-systems-fit-ageing-population

[CR40] Pani-Harreman KE, Bours GJJW, Zander I, Kempen GIJM, van Duren JMA (2021). Definitions, key themes and aspects of ‘ageing in place’: a scoping review. Ageing Soc.

[CR41] Petretto D, Vinci S, Todde I, Piras P, Pistis I, Masala C (2017). Conceptual models of disability and their role in the daily routine of clinical rehabilitation. Rehabilitation Sciences.

[CR42] Pope AM, Tarlov AR, Pope AM, Tarlov AR (1991). A model for disability and disability prevention. Disability in america: Toward a national agenda for prevention.

[CR43] Putnam M (2003). Linking aging theory and disability models: Increasing the potential to explore aging with physical impairment. Gerontologist.

[CR44] Ravenek MJ, Skarakis-Doyle E, Spaulding SJ, Jenkins ME, Doyle PC (2013). Enhancing the conceptual clarity and utility of the international classification of functioning, disability & health: The potential of a new graphic representation. Disabil Rehabil.

[CR45] Reay T, Goodrick E, D'Aunno T (2021). Health care research and organization. Theory.

[CR46] Resnick B, Galik E, Boltz M (2013). Function focused care approaches: literature review of progress and future possibilities. J Am Med Dir Assoc.

[CR47] Romaioli D, Contarello A (2019). Redefining agency in late life: the concept of ‘disponibility’. Ageing Soc.

[CR48] Rostgaard T, Glendinning C, Gori C, Kroger T, Osterle A, Szebehely M, Vabo M (2011). Livindhome: living independently at home: reforms in home care in 9 European countries.

[CR49] Scheidt RJ, Norris-Baker C (2004). The general ecological model revisited: evolution, current status, and continuing challenges. Annu Rev Gerontol Geriatr.

[CR50] Schneidert M, Hurst R, Miller J, Ustün B (2003). The role of environment in the international classification of functioning, disability and health (ICF). Disabil Rehabil.

[CR51] Szanton SL, Xue Q-L, Leff B, Guralnik J, Wolff JL, Tanner EK, Gitlin LN (2019). Effect of a biobehavioral environmental approach on disability among low-income older adults: a randomized clinical trial. JAMA Intern Med.

[CR52] Szanton SL, Leff B, Li Q, Breysse J, Spoelstra S, Kell J, Gitlin LN (2021). CAPABLE program improves disability in multiple randomized trials. J Am Geriatr Soc.

[CR53] Tappenden P, Campbell F, Rawdin A, Wong R, Kalita N (2012). The clinical effectiveness and cost-effectiveness of home-based, nurse-led health promotion for older people: a systematic review. Health Technol Assess.

[CR54] Ustün TB, Chatterji S, Bickenbach J, Kostanjsek N, Schneider M (2003). The international classification of functioning, disability and health: a new tool for understanding disability and health. Disabil Rehabil.

[CR55] Vaz DV, Silva PL, Mancini MC, Carello C, Kinsella-Shaw J (2017). Towards an ecologically grounded functional practice in rehabilitation. Hum Mov Sci.

[CR56] Verbrugge LM, Jette AM (1994). The disablement process. Soc Sci Med.

[CR57] Wahl HW, Fange A, Oswald F, Gitlin LN, Iwarsson S (2009). The home environment and disability-related outcomes in aging individuals: what is the empirical evidence?. Gerontologist.

[CR58] Whitehead PJ, Worthington EJ, Parry RH, Walker MF, Drummond AE (2015). Interventions to reduce dependency in personal activities of daily living in community dwelling adults who use homecare services: a systematic review. Clin Rehabil.

[CR59] Whiteneck G, Field MJ, Jette AM, Martin L (2006). Appendix B: Conceptual models of disability: Past, present, and future. Workshop on disability in america: a new look: summary and background papers.

[CR60] Wiles JL, Leibing A, Guberman N, Reeve J, Allen RE (2012). The meaning of "aging in place" to older people. Gerontologist.

[CR61] Wohlin C (2014) Guidelines for snowballing in systematic Literature studies and a replication in software engineering. Paper presented at the Proceedings of the 18th International conference on evaluation and assessment in software engineering, London, England, United Kingdom. 10.1145/2601248.2601268

[CR62] World Health Organization (1980). International classification of impairments, disabilities, and handicaps: a manual of classification relating to the consequences of disease.

[CR63] World Health Organization (2001). International classification of functioning, disability and health: ICF.

[CR64] World Health Organization (2015) World report on Ageing and Health. Retrieved from Geneva: https://apps.who.int/iris/handle/10665/186463?mode=full

[CR65] World Health Organization (2017) Age-friendly environments in Europe: A handbook of domains for policy action. Retrieved from https://www.euro.who.int/en/publications/abstracts/age-friendly-environments-in-europe.-a-handbook-of-domains-for-policy-action-2017

[CR66] World Health Organization (2020) Decade of Healthy Ageing 2020–2030. Retrieved from https://www.who.int/initiatives/decade-of-healthy-ageing

